# The Synthetic Curcumin Analog HO-3867 Rescues Suppression of PLAC1 Expression in Ovarian Cancer Cells

**DOI:** 10.3390/ph14090942

**Published:** 2021-09-21

**Authors:** Eric J. Devor, Brandon M. Schickling, Jace R. Lapierre, David P. Bender, Jesus Gonzalez-Bosquet, Kimberly K. Leslie

**Affiliations:** 1 Department of Obstetrics and Gynecology, University of Iowa, Carver College of Medicine, Iowa City, IA 52242, USA; brandon.schickling@duke.edu (B.M.S.); jace-lapierre@uiowa.edu (J.R.L.); david-bender@uiowa.edu (D.P.B.); jesus-gonzalezbosquet@uiowa.edu (J.G.-B.); Kimberly-leslie@uiowa.edu (K.K.L.); 2 Holden Comprehensive Cancer Center, University of Iowa Hospitals and Clinics, Iowa City, IA 52242, USA

**Keywords:** ovarian cancer, OVCAR3, ES-2, placenta-specific protein 1 (PLAC1), p53, HO-3867, steric hindrance, reactivator

## Abstract

Elevated expression of placenta-specific protein 1 (PLAC1) is associated with the increased proliferation and invasiveness of a variety of human cancers, including ovarian cancer. Recent studies have shown that the tumor suppressor p53 directly suppresses PLAC1 transcription. However, mutations in p53 lead to the loss of PLAC1 transcriptional suppression. Small molecules that structurally convert mutant p53 proteins to wild-type conformations are emerging. Our objective was to determine whether the restoration of the wild-type function of mutated p53 could rescue PLAC1 transcriptional suppression in tumors harboring certain *TP53* mutations. Ovarian cancer cells OVCAR3 and ES-2, both harboring *TP53* missense mutations, were treated with the p53 reactivator HO-3867. Treatment with HO-3867 successfully rescued PLAC1 transcriptional suppression. In addition, cell proliferation was inhibited and cell death through apoptosis was increased in both cell lines. We conclude that the use of HO-3867 as an adjuvant to conventional therapeutics in ovarian cancers harboring TP53 missense mutations could improve patient outcomes. Validation of this conclusion must, however, come from an appropriately designed clinical trial.

## 1. Introduction

Placenta-specific protein 1 (PLAC1) is a unique 212 amino acid secreted cell surface protein. PLAC1 was initially identified because it is located in a region of the human and mouse X-chromosome that contains other loci suspected of being involved in fetal and placental pathologies [1]. Under normal circumstances, the expression of PLAC1 is almost exclusively limited to the apical surfaces of placental trophoblasts [2]. Placenta-specific expression of PLAC1 has led to its implication in a variety of gestational disorders, including pre-term birth and preeclampsia [3,4,5,6,7,8,9,10,11,12,13,14,15]. In the two decades since PLAC1 was initially reported, many papers have demonstrated its co-opted expression in numerous human cancers [16,17,18,19,20,21,22,23,24,25,26,27,28,29,30,31,32,33,34,35,36,37,38]. In all of these studies, increased PLAC1 expression is linked to increased invasiveness, proliferation and/or aggressiveness, as well as poor prognosis, though the precise mechanisms remain poorly defined. Regardless, it is well established that *PLAC1* is an oncogene in addition to being a fetal–placental gene.

Studies of the regulation of PLAC1 expression identified two promoters, termed P1 (distal), located at exon 1, and P2 (proximal), located at exon 4. Transcripts are produced from both promoters simultaneously in cells that express PLAC1 mRNAs [39,40]. However, in placental tissues, *PLAC1* is predominantly transcribed from the P2 promoter, while in tumors and fetal tissues, *PLAC1* is predominantly transcribed from the P1 promoter. Chen et al. [39] identified RXRα and LXRβ as promoters of P1 transcription, and later, Chen et al. [41] showed in vitro that p53 protein suppresses *PLAC1* transcription by blocking the binding of these transcription factors to the *PLAC1* P1 promoter. We subsequently confirmed the p53 suppression of *PLAC1* transcription in a high-grade serous ovarian cancer (HGSOC) tumor panel [32]. However, in that study, we found that *PLAC1* transcriptional suppression was dependent upon the mutation status of the *TP53* gene. Specifically, only wild-type p53 suppressed *PLAC1* transcription, while any *TP53* mutation appears to permit P1 *PLAC1* transcription. We identified a non-canonical p53 binding site just upstream from the P1 RXRα and LXRβ binding sites. These results led us to propose that P1 *PLAC1* transcriptional suppression is due to steric hindrance when wild-type p53 is bound. Conversely, P1 *PLAC1* transcription in the presence of mutant p53 is permitted due to the failure of mutated p53 to bind and block the RXRα and LXRβ binding sites.

Recognition that mutant p53 permits P1 *PLAC1* transcription in HGSOC tumors and that increased PLAC1 expression in these cancers leads to poor patient outcomes suggested that altering P1 *PLAC1* transcription can be accomplished by targeting p53 itself. *TP53* harbors both missense mutations, which result in single amino acid changes, as well as frameshift/splice site mutations that result in the loss of protein expression. p53 missense mutants can be targeted by molecules that reactivate their wild-type function. One agent that appears to be well suited to this task is the synthetic curcumin analog HO-3867. In the most extensive study to date, the treatment of various cancer cells harboring non-truncating *TP53* mutations with HO-3867 restored the wild-type transcriptional regulation of a number of key p53 target genes [42]. Herein we report that HO-3867 treatment rescues *PLAC1* transcriptional suppression in *TP53*-mutated ovarian cancer cells. Reestablishment of PLAC1 downregulation via p53 reactivation could be used in combination with other therapies to improve outcomes for patients presenting with ovarian carcinomas harboring missense *TP53* mutations.

## 2. Results

### 2.1. HO-3867 Treatment of Ovarian Cancer Cells Restores Suppression of PLAC1 Transcription

Our studies were carried out on two well-characterized ovarian cancer cell lines that contain *TP53* missense mutations, OVACR3 and ES-2. OVCAR3 cells contain the frequent p53 gain-of-function mutant R248Q and were derived from a patient presenting with high-grade serous ovarian cancer, the most common and deadly type. ES-2 ovarian cancer cells are a model of clear-cell ovarian carcinoma and harbor a different p53 binding domain mutation, S241F. While not regarded as a gain-of-function mutant, S241F is classified as pathogenic in COSMIC (https://cancer.sanger.ac.uk/cosmic (accessed on 11 October 2019)) and is a DNA contact mutant overrepresented in ovarian cancers [43].

**Figure 1:**
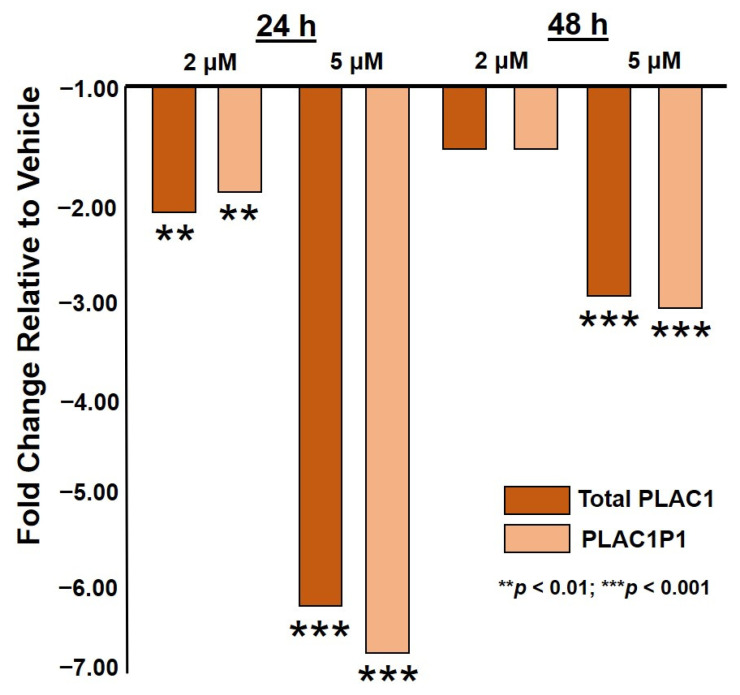
Total PLAC1 and PLAC1P1-specific expression in OVCAR3 ovarian cancer cells treated with 2 µM and 5 µM HO-3867 for 24 h and 48 h.

First, the OVCAR3 cells were exposed to 2 µM and 5 µM HO-3867 for 24 h and 48 h. PLAC1 expression relative to untreated OVCAR3 cells is shown in Figure 1. In all cases, HO-3867-treated cells displayed reduced PLAC1 transcription. Moreover, this suppression was focused at the P1 promoter.

**Figure 2:**
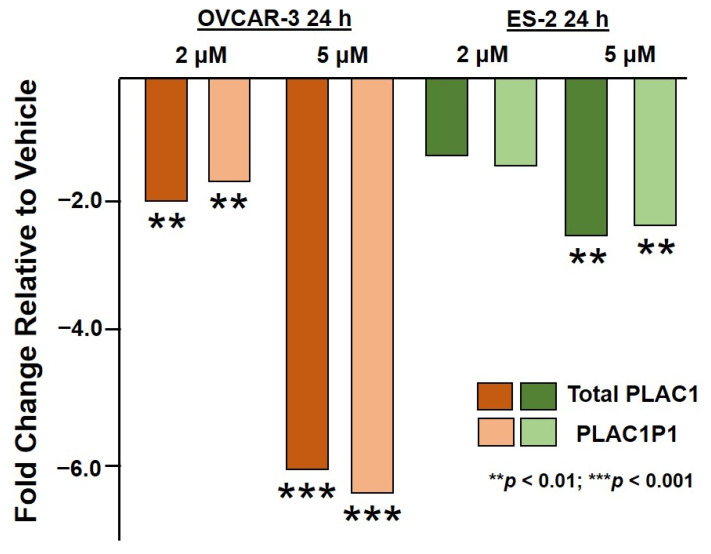
Total PLAC1 and PLAC1P1-specific expression in ES-2 ovarian cancer cells treated with 2 µM and 5 µM HO-3867 for 24 h. ES-2 cells exposed to 2 µM HO-3867 for 48 h did not yield RNA of sufficient quality to perform qPCR, and ES-2 cells exposed to 5 µM HO-3867 for 48 h did not survive. The 24 h results for OVCAR3 cells are shown on the left for comparison.

Treatment of the ES-2 cells with 2 µM and 5 µM HO-3867 for 24 h and 48 h produced a similar, though not as dramatic, rescue of PLAC1 transcriptional suppression (Figure 2). Again, transcriptional suppression appeared to be focused on transcripts originating from the PLAC1 P1 promoter.

### 2.2. HO-3867 Treatment of Both OVCAR3 and ES-2 Ovarian Cancer Cells Results in a Reduction in Proliferation and an Increase in Apoptosis

**Figure 3:**
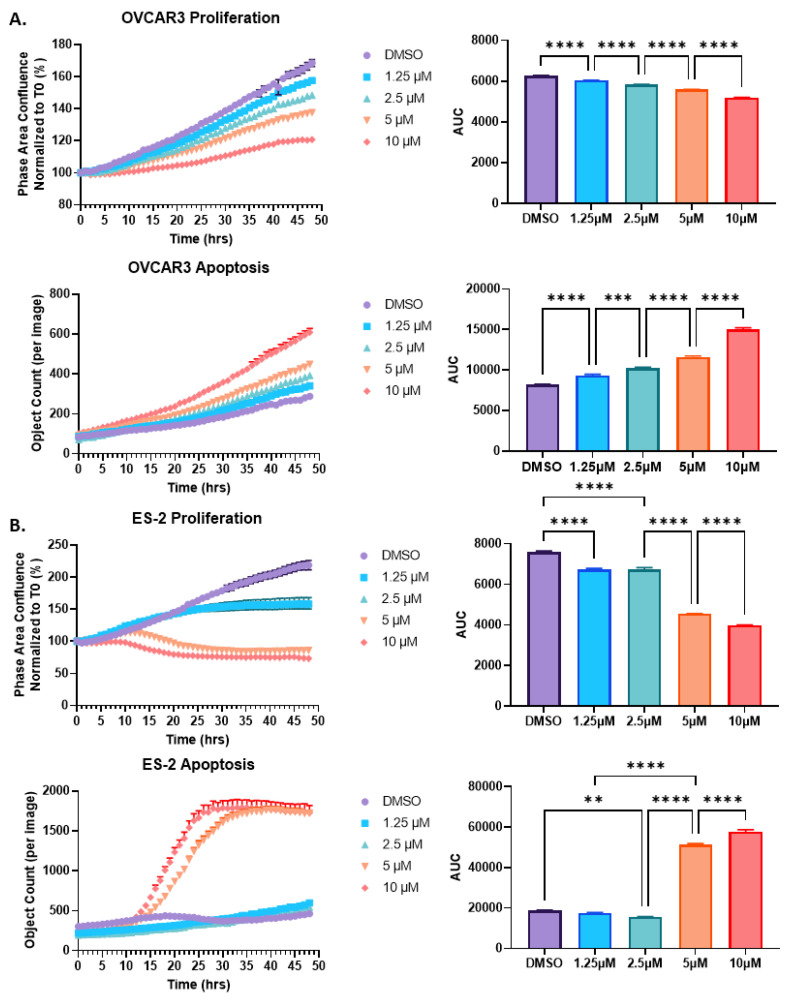
Proliferation and apoptosis in OVCAR3 (**A**) and ES-2 (**B**) cells. Cells were treated with increasing amounts of HO-3867, as shown. Proliferation was measured by phase contrast, and apoptosis was assessed by caspase 3/7 activation. Cells were monitored hourly for 48 h using the Incucyte S3 live-cell imaging platform. Area under the curve (AUC) values were calculated automatically by the Incucyte Software and analyzed via ANOVA. Significance values are indicated as ** *p* < 0.01; *** *p* < 0.001; **** *p* < 0.0001.

Given the importance of p53 in proliferation and apoptosis, we next assessed the functional effect of HO-3867 exposure on both the OVCAR3 and ES-2 cells. Overall, the HO-3867 treatment revealed a dose-dependent decrease in proliferation and a dose-dependent increase in apoptosis in both cell lines (Figure 3). Specifically, treatment of the ES-2 cells with HO-367 showed decreased proliferation at 1.25 µM and 2.5 µM with no change in apoptosis, whereas with higher doses of HO-3867 (5 µM and 10 µM), the ES-2 cells had decreased proliferation and increased apoptosis. Interestingly, HO-3867 had a greater effect on the ES-2 cells compared to the OVCAR3 cells for both proliferation and apoptosis, especially at higher concentrations of HO-3867. The results indicate that HO-3867 is capable of arresting cell growth and inducing cell death through the reactivation of p53 and that the effect of HO-3867 is not only cell-type-specific but also putatively dependent on p53 mutation status.

## 3. Discussion

It has long been recognized that a *TP53* mutation is a molecular vulnerability awaiting therapeutic exploitation [44,45,46,47]. Wild-type p53 has a wide variety of cellular roles, including being a powerful tumor suppressor. Thus, it follows that the re-introduction of wild-type p53 into tumor cells would result in a positive anti-tumor effect [48]. However, attempts to do this have so far proved unsuccessful [49,50]. For example, gene therapy with p53 failed in clinical trials due to its insufficient accumulation in tumor tissue [49]. A promising alternative strategy would be to introduce a compound that is specific for mutant p53 and would also restore wild-type function. To this end, a number of such compounds have been developed, including quinuclidines, 2-sulfonylpyrimidines, zinc metallochaperones and thiosemicarbizones [51]. Some of these are well known, such as COTI-2 [52], PRIMA1 and PRIMA1^met^, now designated as APR-246 [53,54]. Here, we have chosen to assess the less well-known synthetic diarylidenylpiperidone curcumin analog, designated HO-3867, for the purpose of rescuing the suppression of the expression of the oncogene *PLAC1* in ovarian cancer cells harboring a missense *TP53* mutation.

HO-3867 binds p53 at Cys182 and Cys277, creating a “clamp” that holds the β-sandwich in place in a proper DNA-binding configuration while also stabilizing the Zn^++^ binding pocket [42]. The mature p53 protein in this configuration is thus able to function like wild-type p53, including in binding DNA and inducing the transcription of canonical p53 target genes. This was shown by Madan et al. [42] in a panel of 29 human cancer cell lines representing 11 different cancers. These cell lines harbor a total of 26 different missense *TP53* mutations, including six classified as “gain of function” mutants [55]. The effect of exposing these cell lines to HO-3867 was assessed via qPCR expression assays on fourteen known p53 transcriptional target loci [42,56,57]. In the vast majority of these 406 assays (29 cell lines by 14 loci), HO-3867 exposure rescued the appropriate up- or down-regulation of transcription. Indeed, the overall pattern of transcriptional changes was highly significant (5 × 10^−211^) [42]. Our study adds to the literature by providing evidence for the appropriate down-regulation of another p53-regulated gene, PLAC1, as well as demonstrating the effect in ovarian cancer cells, which were not included in the Madan et al. study [42].

**Figure 4:**
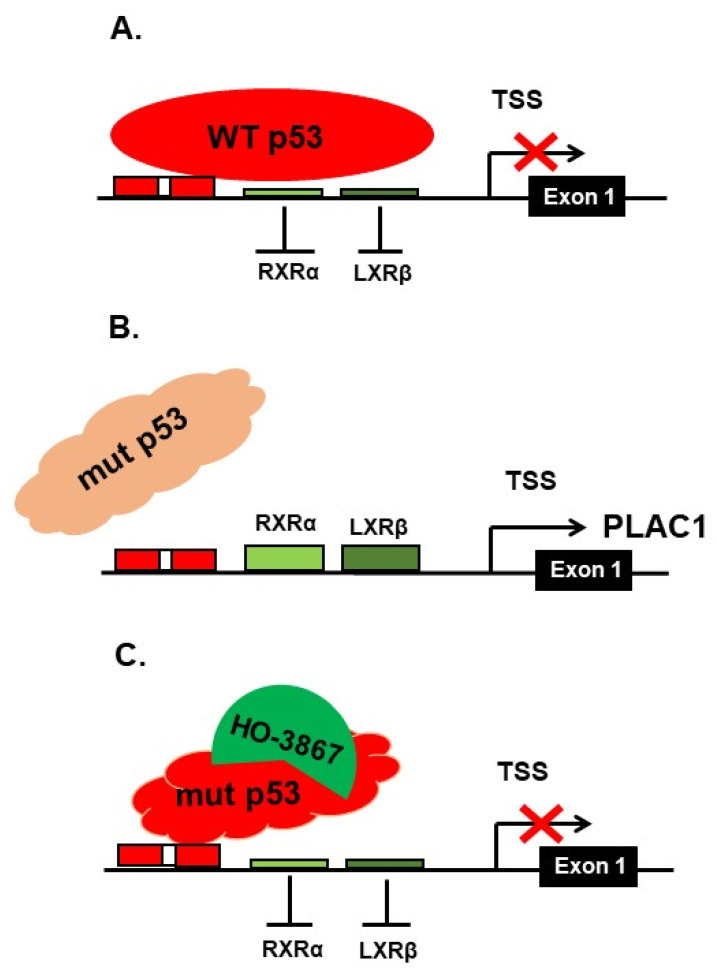
A model of the effect of HO-3867 on p53 binding in the P1 promoter of *PLAC1*. In the top panel (**A**), wild-type p53 freely binds to its binding site, sterically hindering access of the P1 transcription factors RXRα and LXRβ to their binding sites. This has the effect of suppressing *PLAC1* transcription. However, when p53 is mutated, as in the middle panel (**B**), p53 is unable to bind to its binding site, thus freeing the P1 transcription factors RXRα and LXRβ to bind to theirs and promoting *PLAC1* transcription. When exposed to HO-3867, as in the lower panel (**C**), certain p53 mutants are reactivated and will bind to the p53 binding site, once again sterically hindering access of the P1 transcription factors RXRα and LXRβ to their binding sites and hindering *PLAC1* transcription. TSS is the transcription start site.

A model describing the effect of HO-3867 exposure on *PLAC1* transcription in cancer cells harboring DNA-binding domain missense mutations is presented in Figure 4. In this model the P1 promoter is sterically blocked when wild-type p53 binds to its nearby binding site. This leads to reduced *PLAC1* transcription. However, mutant p53 is unable to bind to that site, thus opening up *PLAC1* transcription. Exposure to HO-3867 will, in some mutants, restore wild-type configuration, once again sterically blocking the binding site and reducing *PLAC1* transcription.

Where ovarian cancer has been the focus of the effect of HO-3867 is a series of papers originating from The Ohio State University [58,59,60,61]. In those papers, it has been demonstrated that HO-3867 is cytotoxic to ovarian cancer cells through the induction of apoptosis and the abrogation of cisplatin resistance. Their primary readout was STAT3 protein expression and STAT3 phosphorylation. It should be noted that STAT3 is also a direct transcriptional target of p53 [56], though it remains unclear if the p53 regulation of STAT3 is the mechanism of action of HO-3867. Thus, it is possible that the changes in proliferation and apoptosis in our study may be due to the restoration of p53 regulation of genes in addition to *PLAC1*.

Finally, while it can be argued that the treatment of ovarian cancer patients with HO-3867—preferably as an adjuvant to conventional therapies—could improve outcomes for these patients, its effect is only possible for those cancers that harbor a *TP53* missense mutation. Obviously, nonsense mutants will often result in the truncation of transcripts that are likely to be eliminated by nonsense-mediated RNA decay [62] or another mechanism that would render the cells devoid of p53 protein expression (i.e., p53 null). Further, the small number of *TP53* missense mutations that occur outside the DNA-binding domain will also not be rescued by HO-3867, as the mechanism of HO-3867 action would not, for example, affect p53 tetramerization. However, taking all of the potential exceptions to the effect of HO-3867 exposure on ovarian cancer into account, there remains around 50% of all ovarian cancers that will qualify. Given the fact that the American Cancer Society estimates that there are roughly 22,000 new cases of ovarian cancer in the United States each year, some 11,000 patients could potentially benefit from adding HO-3867 to their treatment regimens. Naturally, the efficacy of using such an adjuvant therapy can only be assessed through an appropriately designed clinical trial.

## 4. Materials and Methods

### 4.1. Cell Culture

NIH:OVCAR3 cells were established from ascites from a 60-year-old patient presenting with a poorly differentiated ovarian adenocarcinoma [63]. We obtained these cells from the American Type Culture Collection (HTB-161) and propagated them in RPMI-1640 media supplemented with 20% fetal bovine serum, 0.01 mg/mL bovine insulin and 1% pen/strep antibiotic at 37 °C and 5% CO_2_.

ES-2 cells were established from primary tumor tissue from a 47-year-old patient presenting with clear-cell adenocarcinoma [64]. We obtained these cells from AddexBio (C0017006) and propagated them in McCoy’s 5A media supplemented with 10% fetal bovine serum and 1% pen/strep antibiotic at 37 °C and 5% CO_2_.

Cell line identities were verified via CODIS DNA profiling at BioSynthesis, Inc. (Lewisville, TX, USA) and by direct *TP53* sequencing. Sequencing confirmed that our OVCAR3 cells harbor the R248Q gain-of-function mutant and that our ES-2 cells harbor the unknown function S241F missense mutant as expected.

### 4.2. HO-3867 Treatment

The HO-3867 reagent was purchased from Cayman Chemical (Ann Arbor, MI, USA, Item No. 21581). Five milligrams of HO-3867 crystals were dissolved in 10.76 mL of DMSO to produce a 1 mM stock solution that was then added to the appropriate cell media to generate the final experimental concentrations.

Depending upon the assay to be carried out, the OVCAR3 and ES-2 cells were seeded in optimum media in 6-well or 96-well culture plates (200,000 cells and 5000 cells per well, respectively) for 24 h prior to the HO-3867 treatment.

### 4.3. RNA Purification and Quality Control

The total cellular RNA was purified from cells using the RNeasy Plus Kit following the manufacturer’s instructions (QIAGEN, Germantown, MD, USA). The RNA yield and purity were assessed in the Genomics Division of the Iowa Institute of Human Genetics (IIHG) using an Agilent Model 2100 DNA Analyzer and a Trinean DropSense 16 spectrophotometer. Samples with an RNA integrity number (RIN) above 7.0 were used for subsequent protocols [65].

### 4.4. Real-Time PCR

Equal-mass RNA aliquots (500 ng) from treated cells were reverse transcribed in the presence of SuperScript III Reverse Transcriptase and an oligo (dT) primer (Thermo Fisher Scientific, Waltham, MA, USA). The resulting cDNAs were then amplified in the presence of Power SYBR Green (Thermo Fisher) on an Applied Biosystems Model 7900HT platform in the Genomics Division of the Iowa Institute of Human Genetics (IIHG) using the locus-specific primer pairs shown in Table 1.

The raw expression Ct values were normalized against 18S rRNA (ΔCt). The fold change relative to the untreated cells was calculated via the conventional ΔΔCt method, where fold change is 2^−ΔΔCt^ [66,67]. The statistical significance of fold changes was assessed via a *t*-test with unequal variances [68]. All experiments were carried out in triplicate.

### 4.5. Cell Proliferation and Apoptosis

Proliferation: The OVCAR3 and ES-2 cells were plated on 96-well plates. Following a 24 h incubation, the cells were treated with increasing amounts of HO-3867. The cells were imaged using the Incucyte S3 live-cell imaging platform (Sartorius AG, Göttingen, Germany) every hour for up to 48 h. The phase-contrast images were processed using masks and filters to determine cell confluence with the Incucyte Analysis Software (2019B Rev3). Proliferation was plotted as percent confluence normalized to time 0.

Apoptosis: The OVCAR3 and ES-2 cells were plated on 96-well plates. Following a 24 h incubation, the cells were treated with increasing amounts of HO-3867 in combination with 5 uM Incucyte Caspase-3/7 Green Dye (Sartorius # 4440). The cells were imaged with the Incucyte S3 fluorescence module every hour for up to 48 h. The fluorescent images were processed with the Incucyte Analysis software (2019B Rev3). Caspase 3/7 activation was plotted as the number of fluorescent objects per image. The graphs were created using GraphPad Prism (v9.1.0). All experiments were carried out in triplicate.

## 5. Conclusions

We have demonstrated that the synthetic curcumin analog HO-3867 successfully rescues the p53- mediated suppression of *PLAC1* transcription in ovarian cancer cells harboring non-truncating *TP53* mutations. As a high expression of PLAC1 is well known to be associated with poor patient outcomes in a variety of cancers, the use of this compound in conjunction with conventional therapies may serve to improve these outcomes.

## Figures and Tables

**Table 1:** Primer pairs used for SYBR Green qPCR in this study. Amplification conditions are 95 °C (10:00); 40 (95 °C (0:15), 60 °C (1:00)). A dissociation curve is run at 95 °C (0:15); 60 °C (0:15); 95 °C (0:15) following the amplification.

Locus	Sequence	Amplicon	Tm (°C) *
*PLAC1*	5′-CACCAGTGAGCACAAAGCCACATT-3	232 bp	60.3
	5′-CCATGAACCAGTCTATGGAG-3′		52.3
*PLAC1P1*	5′-AAACTTACACGAGGAGTCTGTC-3′	371 bp #	57.2
	5′-CTGTGACCATGAACCAGTCTAT-3′	285 bp #	54.2
*18S rRNA*	5′-AACTTTCGATGGTAGTCGCCG-3′	104 bp	57.2
	5′-CCTTGGATGTGGTAGCCGTTT-3′		54.2

* Tm calculated at 1.5 mM MgCl_2_. # Amplicons are from the simultaneously produced P1Long and P1Short transcripts [40].

## Data Availability

Data are contained within the article.
